# The Chinese, Their Present and Future: Medical, Political, and Social

**Published:** 1892-12

**Authors:** 


*The Chinese, their Present and Future: Medical, Political, and Social.* By ROBERT COLTMAN, Jun., M.D. Pp. viii., 212. Philadelphia: F. A. DAVIS. 1891.

This is an American book—American, to some extent, in its language, and American in its plain, practical character and its shrewd common sense. The author was a medical missionary in Shantung, a north-eastern maritime province, of which we in Europe have heard from time to time in connection with the inundations of that erratic, turbulent, and destructive stream, the Hoang-Ho or Yellow River.

Though the form of civilisation is the same throughout the Flowery Land, we learn that there are considerable provincial modifications or varieties of race, of physical and of moral character, and even of language. Dr. Coltman has even acted as interpreter, on several occasions, between northern and southern men, though the written language and character are the same. The northern men are strong, hardy, and enduring, on a dietary of little more than bread, millet-gruel, a kind of coarse macaroni, cucumbers and radishes, and hot water. Shantung is not a tea-growing province, and hot water apparently takes the place of tea among the labouring classes. In a country "where the soil is as porous as a sieve," and where sanitary precautions and the germ-theory are unknown, the drinking-water is of course contaminated; and the Chinese know by experience that no water that has not been boiled is safe for drinking. Good tea cannot be bought in some parts of Shantung under a dollar per pound.

The common characteristics of the people are described in much the same terms as those to which we are accustomed. The Chinese are said to be, generally speaking, industrious and persevering, but procrastinating; imitative, but not inventive; proud, yet given to lying and cheating, and incapable of secrecy. With regard to prostitution, the difference between the Chinaman and the Japanese seems to be like that between the Englishman and the Frenchman. The vice is common everywhere; but the Chinaman is too proud to indulge in it publicly, as is the case in Japan. Female infanticide, the author thinks, is very rare in Shantung, though so common in Southern China; but the female sex is very little esteemed, while male offspring are earnestly desired.

Dr. Coltman is of the anti-opium party, taking a strong view of the pernicious effects of the drug.

The prevailing diseases of Shantung are those of the alimentary tract and of the eyes. Ulcers of the cornea are common: poor diet may be a cause of this, as well as of many of the dyspeptic complaints. Diseases of the lungs and pleura are common along the coast, but not so in some parts of the interior. Acute pulmonary tuberculosis, beginning with hæmoptysis, is occasionally seen; so is a fatal form of leucocythæmia. Dr. Coltman's remarks on stricture of the œsophagus are interesting. It is said to be very common, but often capable of cure or relief. It is attributed to the irritative effects of the wine (or impure spirit) drunk hot; but the author thinks it is usually of syphilitic origin, as most of his cases have improved under the employment of dilatation and of iodide of potassium.

Parasites are extremely common. "Nearly everyone has worms;" and santonine is in high favour.. Dysentery is the most formidable of all diseases to the foreigner; the author's treatment is chiefly remarkable for the absence from it of large doses of ipecacuanha: in the later stages he uses lead and opium, sulphate of copper and opium, cinchona-bark, and enemata.

Dr. Coltman speaks of an anæsthesia cutis unconnected with leprosy. But true leprosy is of very ancient date in China. In Shantung the lepers are not segregated, though the disease is thought to be contagious in some mysterious way. It is regarded as incurable. Our author believes it to be hereditary in most cases, feebly contagious, and inoculable, and he thinks that the previous saturation of the body with syphilis affords a favourable soil for the development of the disease. Accordingly, his internal treatment chiefly consists in the administration of iodide of iron.

There are interesting chapters on missionaries, and on the commercial and political future of China. The book conveys a considerable amount of instruction, and is very prettily illustrated with photo-engravings.

